# Fusing metabolomics data sets with heterogeneous measurement errors

**DOI:** 10.1371/journal.pone.0195939

**Published:** 2018-04-26

**Authors:** Sandra Waaijenborg, Oksana Korobko, Ko Willems van Dijk, Mirjam Lips, Thomas Hankemeier, Tom F. Wilderjans, Age K. Smilde, Johan A. Westerhuis

**Affiliations:** 1 Biosystems Data Analysis, Swammerdam Institute for Life Sciences, University of Amsterdam, Amsterdam, The Netherlands; 2 Department of Human Genetics, Leiden University Medical Center, Leiden, The Netherlands; 3 Department of Endocrinology and Metabolism, Leiden University Medical Center, Leiden, The Netherlands; 4 Division Analytical Biosciences, Leiden/Amsterdam Center for Drug Research, Leiden, The Netherlands; 5 Methodology & Statistics Unit, Institute of Psychology, Leiden University, Leiden, The Netherlands; 6 Research Group of Quantitative Psychology and Individual Differences, KU Leuven, Leuven, Belgium; 7 Department of Statistics, North-West University, Potchefstroom, South Africa; National Research Council of Italy, ITALY

## Abstract

Combining different metabolomics platforms can contribute significantly to the discovery of complementary processes expressed under different conditions. However, analysing the fused data might be hampered by the difference in their quality. In metabolomics data, one often observes that measurement errors increase with increasing measurement level and that different platforms have different measurement error variance. In this paper we compare three different approaches to correct for the measurement error heterogeneity, by transformation of the raw data, by weighted filtering before modelling and by a modelling approach using a weighted sum of residuals. For an illustration of these different approaches we analyse data from healthy obese and diabetic obese individuals, obtained from two metabolomics platforms. Concluding, the filtering and modelling approaches that both estimate a model of the measurement error did not outperform the data transformation approaches for this application. This is probably due to the limited difference in measurement error and the fact that estimation of measurement error models is unstable due to the small number of repeats available. A transformation of the data improves the classification of the two groups.

## Introduction

Over the last decades, metabolomics has become an indispensable tool that has provided a wealth of information on biological systems. To get a better grasp of the underlying biochemical processes, combining data from different metabolomics platforms can contribute greatly. Data of each platform provides information on different parts of the metabolism, and by combining the data sets they may complement each other.

The combination of data sets is sometimes referred to as data integration or by data fusion. The difference between these approaches is not well defined. Data integration methods are qualitative approaches that collect measurements of different origins and puts them on a well-defined scaffold, e.g. a metabolic network. Data integration thus consider the identity of the variables in the different data sets. E.g. gene expression data, proteomics and metabolomics data can be integrated by placing their intensity values at the corresponding position in the metabolic network. The identity of the different variables of different sources is taken into account when collecting the data. When a metabolite is measured on two platforms, both variables are linked to the same position on the network. Thus for data integration, network information and ontologies on the meaning of the variables are necessary.

Data fusion methods are quantitative methods that combine multiple data sets that are measured on the same set of samples. The models that are developed are based mainly on correlations between the variables. In data fusion methods, the identity of the variables of the data sets is not directly used in the analysis. Only after the models have been developed, the identity of the variables can be used when interpreting the data analysis results. In this paper the focus is fully on data fusion methods.

Several classification methods exist for high dimensional data [1], e.g. principal component discriminant analysis (PCDA)[2], and partial least squares discriminant analysis (PLS-DA)[3], both of which are frequently used in the field [4,5]. However, these standard methods cannot handle complementary data obtained from multiple platforms. A commonly used method to fuse data from multiple platforms, to reveal their underlying relationships, is simultaneous component analysis (SCA)[6–8]. While useful for certain questions, it does not specifically classify or make predictions about class membership. To overcome this bottleneck, we propose to incorporate SCA into PCDA. This new method, simultaneous component discriminant analysis (SCDA), will allow for proper discrimination between classes by fusing data from different platforms.

The analysis of fused metabolomics data from different platforms however is not straightforward. One of the issues is that most multivariate data analysis methods, and also SCDA, are limited by the fact that homoscedastic error is assumed. In metabolomics, many error sources result in a measurement error variance that is proportional to the measured intensity. This is the reason why relative standard deviations (RSD) are often used to quantify the quality of a metabolomics platform[9]. In more advanced approaches, the measurement error variance is proportional to the intensity level at large intensity levels but constant at low intensity levels [10,11]. Heteroscedastic and even correlated error structures can lead to incorrect estimation of standard errors of discrimination coefficients and thus making incorrect assumptions about variable importance in discrimination models.

Several methods have been applied successfully to remove the non-constant measurement error variance. Transformation of the data is a well- known method to stabilize the variability of the data. The most common transformation approaches are the square root and the log transformation[12,13]. Both methods however, do not take a constant measurement error variance into account for low concentration values. The generalized log (glog) transformation works better in cases where the measurement error stays stable at low intensities, but increases for higher intensities. Generalized log-transformation (glog) of metabolomic data was used to make multivariate classification more effective.

Maximum likelihood scaling (MALS) [14] also takes the measurement errors into account. It uses a maximum likelihood principal component analysis to filter out measurement noise within the data; it down-weights measurements with higher uncertainty, i.e. higher measurement error variances. Both data-transformation and MALS filtering approaches are methods that are applied before data analysis.

We also introduce a third approach to deal with the non-constant measurement error variance. The SCDA method will be extended by down-weighting unreliable data through Maximum Likelihood Fusion. A weighted SCA method is used to obtain unbiased principal components which will successfully be used for the classification. The weights in the weighted SCA are obtained from estimates of the measurement error variance for each measured metabolite level. Such estimates usually come from repeated analysis of quality control samples; however, the quality control sample does not cover a wide range of the metabolite levels. Instead in this approach sample replicates are used for a good characterization of measurement errors of the study. They include both the uncertainty of the analytical method as well as the uncertainty of the sample work-up, and they do cover a large range of metabolite concentration. As multiple samples are replicated in a study, measurement error variance can be estimated at different levels of all metabolites.

We explore the effect of the variance stabilization methods on the data, how well they are able to produce a homoscedastic error variance. Furthermore, we study whether the transformed data is better able to discriminate between the two groups. The error variance stabilization methods are divided into 3 method groups: (1) transformation, (2) filtering, and (3) modelling. The methods are all applied to two metabolomics platforms (lipids and amino acids) obtained from healthy obese and diabetic obese individuals qualified for gastric bypass surgery.

The article is organized as follows. In the Methods section first PCDA and SCA are introduced. Then, the three method groups for correcting heterogeneous error variances are explained, as well as the Rocke-Lorenzato error model. Finally, the metabolomics data of the gastric bypass study and its pre-processing is discussed. The Results section shows the variance stabilization and the classification results.

## Methods

### 2.1. Principal component discriminant analysis

Principal component discriminant analysis (PCDA) [2] is a discrimination method, ideal for high dimensional data [15]. It is used in cases where normal linear discriminant analysis (LDA) fails, i.e. in the presence of multicollinearity and/or high dimensional data. It is a two-step method that summarizes the data into multiple principal components, and additionally performs LDA on the first *R* principal components.

Consider a platform measuring *J* metabolites has been used to measure two groups of *I*_*1*_ and *I*_*2*_ individuals (*I* = *I*_*1*_+*I*_*2*_) then data block **X** (*I* x *J*) containing all these measurements can be subjected to a principal component analysis.

$$X=TP^{T}+E$$ (Eq 1)

Here **T** (*I* x *R*) is the score matrix, **P** (*J* x *R*) the loading matrix, and **E** (*I* x *J*) the residual matrix for the first *R* principal components. **T**_**1**_ (*I*_1_ x *R*) is that part of **T** that contains the score values for the individuals in group 1 and likewise **T**_**2**_ (*I*_2_ x *R*) contains the scores for the individuals in group 2. In the second step, LDA tries to find a linear combination of the first *R* principal component scores such that the between class difference is maximal compared to the within class difference. That is, a weight vector **β** is estimated that maximizes

$$G(\beta )=\frac{\beta^{T}\Sigma_{B}\beta }{\beta^{T}\Sigma_{W}\beta }$$ (Eq 2)

where $\Sigma_{B}=({\bar{T}}_{1}-{\bar{T}}_{2}){\left({\bar{T}}_{1}-{\bar{T}}_{2}\right)}^{T}$ and $\Sigma_{W}=\sum_{g=1,2}\sum_{t_{i}\in T_{g}}(t_{i}-{\bar{T}}_{g}){\left(t_{i}-{\bar{T}}_{g}\right)}^{T}$ are the between class and within class scatter matrix of the score matrices **T**_**1**_ and **T**_**2**_ and ${\bar{T}}_{1},{\bar{T}}_{2}$ are the mean of **T**_**1**_ and **T**_**2**_ respectively. **t**_***i***_ is the score vector of an individual *i*. The offset value in the PCDA model equals $\beta_{0}=\frac{-1}{2}{\left({\bar{T}}_{1}+{\bar{T}}_{2}\right)}^{T}\beta$. Individuals with **t**_***i***_^T^**β** > *β*_0_ are predicted to belong to group 1, and when **t**_***i***_^T^**β** < *β*_0_ they are predicted to belong to group 2. **β** contains the weights for each principal component to provide the best classification. To obtain information on weights in terms of the originally measured metabolites this direction is pre-multiplied by the loadings **P**, ***b***_*PCDA*_ = **Pβ**.

### 2.2. Simultaneous component analysis

Simultaneous component analysis (SCA) [16,17], an extension of PCA, is capable of combining multiple data blocks such that similar individual characteristics contained in different data blocks can be unveiled. If *K* data blocks ***X***_*k*_ share the same object mode with *I* individuals and *J*_*k*_ variables, then the SCA decomposition is as follows:

$$X_{k}=TP_{k}^{T}+E_{k}$$ (Eq 3)

with **T** (*I* x *R*) the score matrix for *R* components shared by all *K* data blocks, **P**_*k*_ (*J*_*k*_ x *R*) the loading matrices, one for each data block, and **E**_k_ (*I* x *J*_*k*_) error matrices. The loadings and score matrices are obtained similar to normal PCA, when all *K* data blocks are fused into one concatenated data block **X**_c_ = [**X**_1_ … **X**_k_ … **X**_K_], with size $(I\;x\;\sum_{k=1}^{K}J_{k})$, with the accompanying loading matrix $P_{c}={\left[P_{1}^{T}\ldots P_{k}^{T}\ldots P_{K}^{T}\right]}^{T}$, of size $(\sum_{k=1}^{K}J_{k}\;x\;R)$. This specific approach of combining multiple blocks in a simultaneous component model is called the SCA-P model[18].

The scores **T** of the SCA can be used in an LDA step similar as was presented for the PCDA model. This method is called simultaneous component discriminant analysis (SCDA).

As PCA focuses on describing maximum variance of the complete data, the scores and loadings in an SCDA model clearly depend on the variances and sizes of the different data blocks. Methods such as block scaling can be used to reduce the effect of variable block size and sum of squares per data block [19].

### 2.3. Correcting for measurement error

An underlying assumption of PCA and SCA is that the errors have equal variance throughout the whole data matrix. In an SCA model for multiple data sets obtained from different platforms, this assumption extends to the situation that each data block has measurement errors around the true values that are considered of the same size, i.e. the homoscedastic measurement error variance assumption. In many cases this assumption is not valid and various approaches have been used to correct for this issue.

#### 2.3.1. Transformation

Log-transformation is a popular and quick method to reduce the large error variance for large values, however, it has the tendency to inflate the variance of values near zero [20]. The generalized log (glog) transformation stabilizes measurement variance over the full range of the data, while it takes into account that for low values the measurement errors are constant but increases for higher intensities [21]. Rocke and Lorenzato [10] assume that the intensity level can be modelled as:

$$x=\mu e^{\eta }+\varepsilon$$ (Eq 4)

where *x* is the measured concentration, *μ* is the true concentration level, and *η* and *ε* are normally distributed error terms, with mean 0 and variance $\sigma_{\eta }^{2}$ and $\sigma_{\varepsilon }^{2}$, respectively. So at high values of *μ*, the measurement error variance increases as a function of *μ*. At low concentrations, when *μ* approaches zero, the variance is stable at $\sigma_{\varepsilon }^{2}$. This kind of data can be transformed using the generalized log (glog) transformation. Moreover, Purohit et al.[13] showed that glog transformation of metabolomics data makes multivariate classification more effective. For glog-transformation, the measured intensity level *x* is transformed as follows

$$g(x)=\ln\left(x+\sqrt{{\left(x\right)}^{2}+\lambda }\right)$$ (Eq 5),

with *λ* being a tuning parameter that can be optimised using repeated measurements with a maximum likelihood method [22]. The addition of the *λ* parameter in the glog function reduces the measurement error variance for low values of *x* to make the error close to homoscedastic.

Additionally, the square root transformation of the data is explored, since it is known to help in problems where the measurement errors increase with increasing intensity [12]. The square root transformation expects a smaller increase of error variance than the log transform. Furthermore, it gives fewer problems for very small values and zeros in the data.

#### 2.3.2. Filtering

Maximum likelihood scaling (MALS) [14] is a two-step procedure that filters out heteroscedastic noise using a weighted PCA (maximum likelihood PCA). The weighted PCA weights each observation proportional to the reciprocal of its measurement error variance, i.e. it minimizes the weighted residual sum of squares S^2^,

$$S^{2}=\min_{T_{R},P_{R}}\sum_{i=1}^{I}\sum_{j=i}^{J}\frac{{\left(x_{ij}-{\hat{x}}_{ij}\right)}^{2}}{\sigma_{ij}^{2}}=\underset{T_{R},P_{R}}{min}{\left\|W\circ (X-\hat{X})\right\|}^{2}$$ (Eq 6)

where $\hat{X}=T_{R}{P_{R}}^{T}$, for *R* principal components, and **W** is the weight matrix with $w_{ij}=\frac{1}{\sigma_{ij}}$, and $\sigma_{ij}^{2}$ the variance of the measurement error for metabolite *j* at the level measured for individual *i*. After this filtering step is performed on each data block separately, the second step, namely (auto-)scaling can be done without the risk of noise amplification and increase of heteroscedasticity of the data.

#### 2.3.3. Modelling with Maximum Likelihood Fusion

SCA assumes a homoscedastic error-function. To ensure compatibility with metabolomics data, for which measurement error variances are known to depend on measurement levels, and to overcome non-constant error variance of the different metabolites, a maximum likelihood version of SCA is used here. This approach weights each observation proportional to the reciprocal of its measurement error variance, i.e. it minimizes the weighted sum of squares S^2^ of all *K* data blocks simultaneously,

$$S^{2}=\min_{T_{R},P_{\mathrm{Rk}}}\sum_{k=1}^{K}\sum_{i=1}^{I}\left(\sum_{j=1}^{J_{k}}\frac{{\left(x_{ij_{k}}-{\hat{x}}_{ij_{k}}\right)}^{2}}{{\sigma_{ij}^{2}}_{k}}\right)=\min_{T_{R},P_{\mathrm{Rk}}}\sum_{k=1}^{K}{\left\|W_{k}\circ (X_{k}-{\hat{X}}_{k})\right\|}^{2}$$ (Eq 7)

where $\hat{X_{k}}=T_{R}{P_{Rk}}^{T}$, for *R* principal components, and **W**_*k*_ is the weight matrix with $w_{ij_{k}}=\frac{1}{\sigma_{ij_{k}}}$, and $\sigma_{ij_{k}}^{2}$ the variance of the measurement error of individual *i* for metabolite *j* of data block *k*.

Minimization of the weighted sum of squares can be accomplished using the MILES maximum likelihood principal component analysis algorithm of Bro et al. [23], which is also used in the filtering step. The difference between the filtering and the modelling method is that the filtering is performed on each data block separately before fusion, thus SCDA is performed on the 'noise-free' data. In the modelling method, the data blocks are fused and additionally weighted within the SCDA.

### 2.4. Measurement error structure model

Both in the filtering as well as in the modelling procedure a weight matrix has to be determined, which exists of the reciprocal of the measurement error variance for measurement on each metabolite. To correct for differences in measurement error variance, an optimal estimated error variance structure is essential. In metabolomics data the measurement error typically increases with measured intensity level [11]. This typical error structure is best described by the Rocke-Lorenzato model [10], which assumes a constant error variance for small intensities and a multiplicative variance for higher intensities. Here, we follow the approximation by van Batenburg et al. [11] of that error model which has a constant part and a part depending on the concentration. The measurement error variance $\sigma_{ij_{k}}^{2}$ is defined as:

$$\begin{array}{lll} \sigma_{ij_{k}}^{2}={\left(\sigma_{Add\;j_{k}}\right)}^{2} & if & \;\mu_{ij_{k}}\leq \alpha_{Mult\;j_{k}}\\ \sigma_{ij_{k}}^{2}={\left(\sigma_{Add\;j_{k}}\right)}^{2}+{\left(\sigma_{Mult\;j_{k}}\right)}^{2}{\left(\mu_{ij_{k}}-\alpha_{Mult\;j_{k}}\right)}^{2} & if & \;\mu_{ij_{k}}>\alpha_{Mult\;j_{k}} \end{array}$$ (Eq 8)

For different values of $\alpha_{Mult\;j_{k}}$, both ${\left(\sigma_{Add\;j_{k}}\right)}^{2}$ and ${\left(\sigma_{Mult\;j_{k}}\right)}^{2}$ are estimated in a two step procedure. In the first step ${\left(\sigma_{Add\;j_{k}}\right)}^{2}$ is estimated for values that are smaller or equal to the cut-off $\alpha_{Mult\;j_{k}}$, i.e. $\mu_{ij_{k}}\leq \alpha_{Mult\;j_{k}}$. In the second step ${\left(\sigma_{Mult\;j_{k}}\right)}^{2}$ is estimated for data where $\mu_{ij_{k}}>\alpha_{Mult\;j_{k}}$, with ${\left({\hat{\sigma }}_{Add\;j_{k}}\right)}^{2}$ obtained from step 1. The best model (with optimal $\alpha_{Mult\;j_{k}}$’s, ${\left(\sigma_{Add\;j_{k}}\right)}^{2}$’s and ${\left(\sigma_{Mult\;j_{k}}\right)}^{2}$’s) is the one that minimizes the sum of the squared difference between the estimated and measured variance, that is $\sum_{i=1}^{I}{\sum_{j=1}^{J_{k}}\left(\sigma_{ij_{k}}^{2}-{\hat{\sigma }}_{ij_{k}}^{2}\right)}^{2}$. To reduce the effect of large outliers, the model parameters are estimated via robust regression using the Matlab *robustfit* function with the *‘huber’* weight function and the default tuning constant.

The Rocke-Lorenzato model is estimated per metabolic group *g* (*g = 1*, *…*, *G*; the amine and 9 lipid groups). Thus $\sigma_{{Add}_{g}},\sigma_{{Mult}_{g}}\;and\;\alpha_{{Mult}_{g}}$ are estimated from $\sigma_{dj_{k}}^{2}$ and $\mu_{dj_{k}}$ for *d* = *1*,*…*, *D* duplicates in the study using all variables *j*_*k*_ that belong to group *g*.

Besides the Rocke-Lorenzato model we also used the median error variance per metabolite to weigh the residuals independent of their measured intensity, to study the effect of the weight matrix in the maximum likelihood fusion model.

### 2.5. Data

The data contains a total of 61 individuals that can be subdivided into 3 groups:

a) 30 lean controls
b) 16 healthy obese individuals, on the list for gastric bypass
c) 15 diabetic obese patients, measured before and 1 to 4 months after gastric bypass

Within each group a number of sample replicates were measured, 4 in the control group (a), 4 in the healthy obese group (b), and 7 in the diabetic obese group with paired data (c), of which 3 before and 4 after treatment (though not from the same individual). A pooled quality control (QC) sample is used to monitor possible instrumental drift.

For each individual 43 amino acids were measured (LC-MS) and their intensity levels are determined by means of an internal standard. Not each metabolite had a unique internal standard; some internal standards were used to standardize multiple metabolites. Injection replicates were averaged. Only a single measurement batch was needed to obtain the data, in which also 19 quality control samples were measured.

In addition to the amino acids, 185 lipids were measured (LC-MS). Here a single internal standard was used per lipid-class. There are nine lipid-classes: triradylglycerolipids (TG), diradylglycerolipids (DG), ceramides (Cer), sphingomyelins (SM), glycerophosphocholines (PC), glycerophosphoethanolamines (PE), lysophosphatidylethanolamine (LPE), lysophosphatidylcholine (LPC), and cholesteryl ester (CE). No injection replicates were determined. A single measurement batch was sufficient to measure all samples and additionally 11 quality control samples.

For both the amino acid and the lipid platform, QC samples were used to correct for potential instrumental drift using the QC correction approach [24]. Intensity levels under the limit of detection were replaced by their QC corrected value. Finally, variables had to have at least 80% measured values above the lower level of detection within one of the before or after groups of data (c). The final data set contained 43 amino acids and 165 lipids.

The goal of this research was to explore the different approaches of correcting for nonconstant measurement errors in the fusion of metabolomics data from different platforms, and whether such a correction can contribute to a more precise discrimination. Therefore, we selected a small part of the data, i.e. the 16 healthy obese patients (group b) and the 15 diabetic obese patients before gastric bypass (group c), both measured at the lipid and amino acid platforms. For the determination of the measurement error structure and the GLOG-transformation all 15 sample replicates were used of group (a), (b), and (c); i.e., we assume that the measurement error model does not depend on the group of individuals.

### 2.6. Pre-processing of the data

For each of the three methods, the data was pre-processed. For the transformation methods, the data was either centered or autoscaled after the transformation. Then blockscaling was applied by scaling each block to a *Frobeniu*s norm equal to 1. MILES [23] with a centering step was used for the weighted PCA in MALS and also in the Maximum Likelihood Fusion model. After MALS filtering, the data was either only centered or autoscaled, after which again a blockscaling was applied to give blocks equal weight after filtering. In the transformation and filtering method, the blockscaled data were next subjected to a SCDA. In the Maximum Likelihood Fusion modelling, the two data sets were autoscaled and blockscaled before the modelling step.

### 2.7. Prediction of class membership for new samples

The class prediction for new samples and also in a cross-validation context (see section 2.8) depends on the method used. Therefore, there is a training set of which scaling parameters (e.g. mean and standard deviation of each metabolite) and model parameters (e.g. discrimination coefficients for each metabolite) are obtained and a test set on which these scaling and model parameters are applied.

For the transformation methods, new data is first transformed before it is subjected to the model of the transformed data. Transformations such as log and square root transformations are performed on the data directly and do not need information from the training set. Except for the glog transformation,

$$g(x_{new})=\ln\left(x_{new}+\sqrt{{\left(x_{new}\right)}^{2}+\lambda }\right)$$

where the λ value for each metabolite is obtained from the glog fitting of the training data. Then g(**x**_**new**_) can be used in the model obtained from the training data.

For scaling methods and centering, scaling parameters are obtained from the training set and applied to the test set.

For the MALS filtering a weighted PCA is applied on the training data using weights for each sample i for variable j_k_ defined as $w_{ij_{k}}=\frac{1}{\sigma_{ij_{k}}}$, i.e. the weight for each element in the data is defined to be the inverse of the standard deviation for that specific element. The standard deviation can come from the Rocke Lorenzato model of the error variance for each metabolite or they are defined as the median error variance for each metabolite. The weighted PCA model parameters are then applied to the test set to filter the heteroscedastic variance from the data. The MALS filtering is also applied in a cross validation approach such that the filtering of each sample is based on weighted PCA model estimates obtained from the other samples. The MALS filtered data are used in a normal PCDA cross validation procedure as discussed in section 2.8. After filtering a PCDA model is applied to the data in a cross model validation approach to prevent overfitting.

Finally, for the modelling approach a weighted PCDA modelling is applied in a cross model validation approach. The model parameters are obtained from the training set and applied to the test set for prediction of class membership.

In all approaches the number of components was not optimized in each cross validation round but always fixed to 3, 5, or 7 components as can be found in Table 1. In this way we can also learn how much the model is effected by the number of selected components.

**Table 1:** Average number of misclassifications.

Method	LV = 3	LV = 5	LV = 7
**Raw Center**	**10.7**	**10.9**	**10.7**
**Raw Auto**	**9.8**	**9.2**	**9.1**
**SQRT Center**	**11.2**	**9.4**	**8.1**
**SQRT Auto**	**9.9**	**9.1**	**9.9**
**Log Center**	**11.8**	**13.0**	**10.8**
**Log Auto**	**10.0**	**8.9**	**9.9**
**Glog Center**	**10.2**	**11.8**	**9.9**
**Glog Auto**	**9.7**	**8.4**	**9.4**
**MALS RL Center**	**14.7**	**14.7**	**12.3**
**MALS RL Auto**	**10.8**	**10.0**	**9.9**
**MALS MED Center**	**12.2**	**13.0**	**13.3**
**MALS MED Auto**	**11.5**	**13.6**	**11.7**
**Weighted MED**	**11.9**	**9.6**	**11.8**
**Weighted RL**	**12.9**	**10.0**	**11.2**

Average number of misclassifications using (W)SCDA methods with different methods for measurement error variance stabilization methods.

### 2.8. Validation

Cross-validation is used to define the classification errors for each of the approaches used. The data was split in 8 parts, where each part contained 2 healthy obese and 2 diabetic obese individuals (except for the last part that only contained a single diabetic obese individual). 7 parts (training set) are used to train the SCDA model which is then used to predict the class of the last part (test set). This is repeated until each individual has been left out in the test set once. The number of incorrectly predicted samples is calculated. A cross model validation procedure was used to prevent overfitting of the classification models. In cross model validation, a subset of the data is left out and in no way used to define the model. The remainder of the data is used to define the model with all model parameters such as the number of components. Only the final model is used to predict the class membership of the left out samples. This is repeated until each sample has been left out once. This procedure is known for its unbiased classification error. If an optimal number of PCDA components is required then this should also have been performed in a cross model validation approach where the training set is used to find the optimal model dimension and the final model parameters.

This cross validation procedure is repeated 25 times with each time different combinations of test set samples, to make sure the results are consistent and not due to chance effects. The average number of misclassifications is given as the result of the classification for a specific pre-processing, filtering or modelling approach.

Within the cross validation procedure, the transformations did not have to be repeated for each new training set as the transformation does not depend on other samples. For the MALS filtering, a new MALS filtering is applied for each training-set as the MALS filter depends on the specific samples in the training set. This model is then used to estimate the scores for the test set samples. Similarly, the weighted SCA model is also calculated newly for each training set.

In this cross validation procedure, we compared the performance for models with 3, 5 or 7 components. The importance of each metabolite is averaged based on the 8x25 models.

### 2.9. Software

Correction of potential instrumental drift using the QC measures [24], was performed in MatLab [25]. Further analysis was performed in Matlab, for PCA the *svd* command is used, linear discriminant analysis was performed using the *classify* function; block-scaling was applied by scaling each matrix by its *Frobenius* norm. Robust regression was performed using *robustfit* with Huber weight function with default tuning constant. *GLOG-*transformations were estimated using the Matlab toolbox described in Parsons et al. [21] Weighted PCA is performed using the MILES Toolbox for Matlab [23].

## Results

### 3.1. Determination of error structures and the effect of transformations

**Fig 1:**
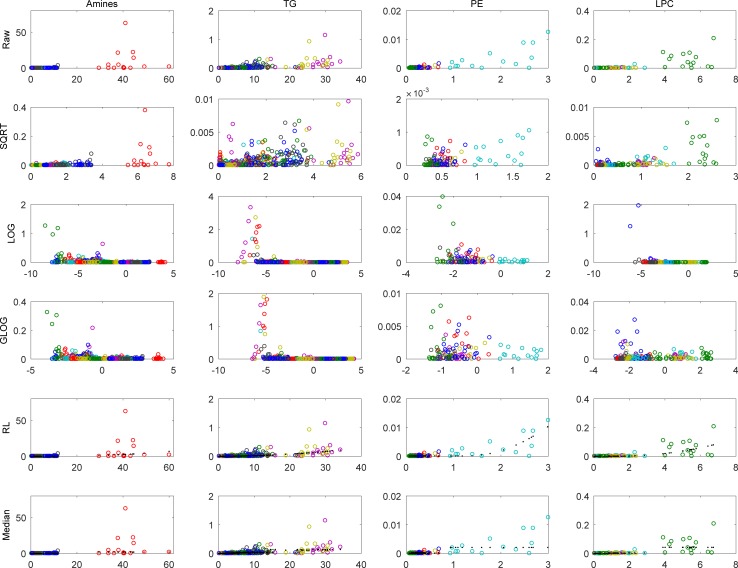
Measurement error variance. Measurement error variance (y-axis) as a function of Mean Ratio (X-axis) for the Amines and three lipid groups (TG, PE, LPC) for the raw data and after SQRT, LOG and GLOG transformation, and the Rocke-Lorenzato (RL) estimates (in black) of the measurement error variance, and the median errors. The color in each column represents a metabolite and circles of the same color are obtained from different samples of which the error variance is estimated from the replicated analyses of that sample.

For further analysis the measurement error structure and the optimal GLOG transformations of the data have to be determined. This was done on the 15 sample replicates. Fig 1 shows the error structure of the amines and a selected number of lipids classes (TG, PE, and LPC). In the first row (raw data) are the estimated error variances on the y-axis as a function of the mean concentration (x-axis) for the amines and the three indicated lipid classes. In each subplot, different colours indicate different metabolites (amines or lipids). As 15 samples were used for the estimation of the error variance model, 15 circles of each colour are observed. For each sample the estimated variance is plotted versus the mean of the two replicates. It can be seen that the levels of the amines and the lipids is rather different, even within their specific classes. In general, the error variance increases for amines and lipids with higher levels. For a single lipid, only the cyan circles in the PE class show a clear increase in error variance for larger lipid levels. The other rows in Fig 1 show the same data after transformation (SQRT, LOG, and GLOG) or they show an estimated model of the error variance (Rocke-Lorenzato and Median error model). The other 6 lipid classes have comparable patterns.

#### 3.1.1.Transformation

Transforming the data using a SQRT or (G)LOG scale is a known method to reduce the measurement error for higher ratios. Fig 1 shows the effect of SQRT, log-transformation and g-log-transformation on the amine and lipid classes. For all transformations the variance in the higher ratios decreases. For the LOG transform there is an increase in variance of the measurement errors for the small ratios. As expected, this effect is (slightly) less for the GLOG-transformed data. The SQRT transformation seems to transform the error variances to a more homoscedastic level.

#### 3.1.2. Measurement error structures

For the given data, increased measurement levels do not seem to result in an observed increase in measurement errors when examining each metabolite separately. Only the lipid represented by the cyan circle in the PE class shows increased variance for increasing mean ratio levels. This might be due to low levels or limited ranges for the measured metabolites. Consequently, a measurement error model with a fixed variance per metabolite may be a better model for our data set. To reduce the effect of large outliers, the median variance per metabolite was also taken for the error structure. Thus $x_{ij_{k}}∼N(u_{ij_{k}},\sigma_{j_{k}}^{2})$, the error model is independent of the measured level of individual (*i*), and it only depends of the measured metabolite (*j*_*k*_) (Fig 1; row 6, Median). The modelled variance is indicated with black dots.

For a good estimation of the Rocke-Lorenzato model, sufficient measurements are necessary. Therefore, we decided to combine all metabolites from the amine platform to estimate the model parameters. For the lipid platform there is a natural segmentation of the lipids into 9 lipid classes, indicated earlier. For all lipids in a lipid class the same internal standard was used. We decided to estimate a Rocke-Lorenzato model for each lipid class, and also for the amine class. As a result, while unnoticed in the error structure per metabolite, we can now clearly observe an increase in the measurement error over a larger range due to the increased level (Fig 1; row 5, RL).

### 3.2. Data exploration

**Fig 2:**
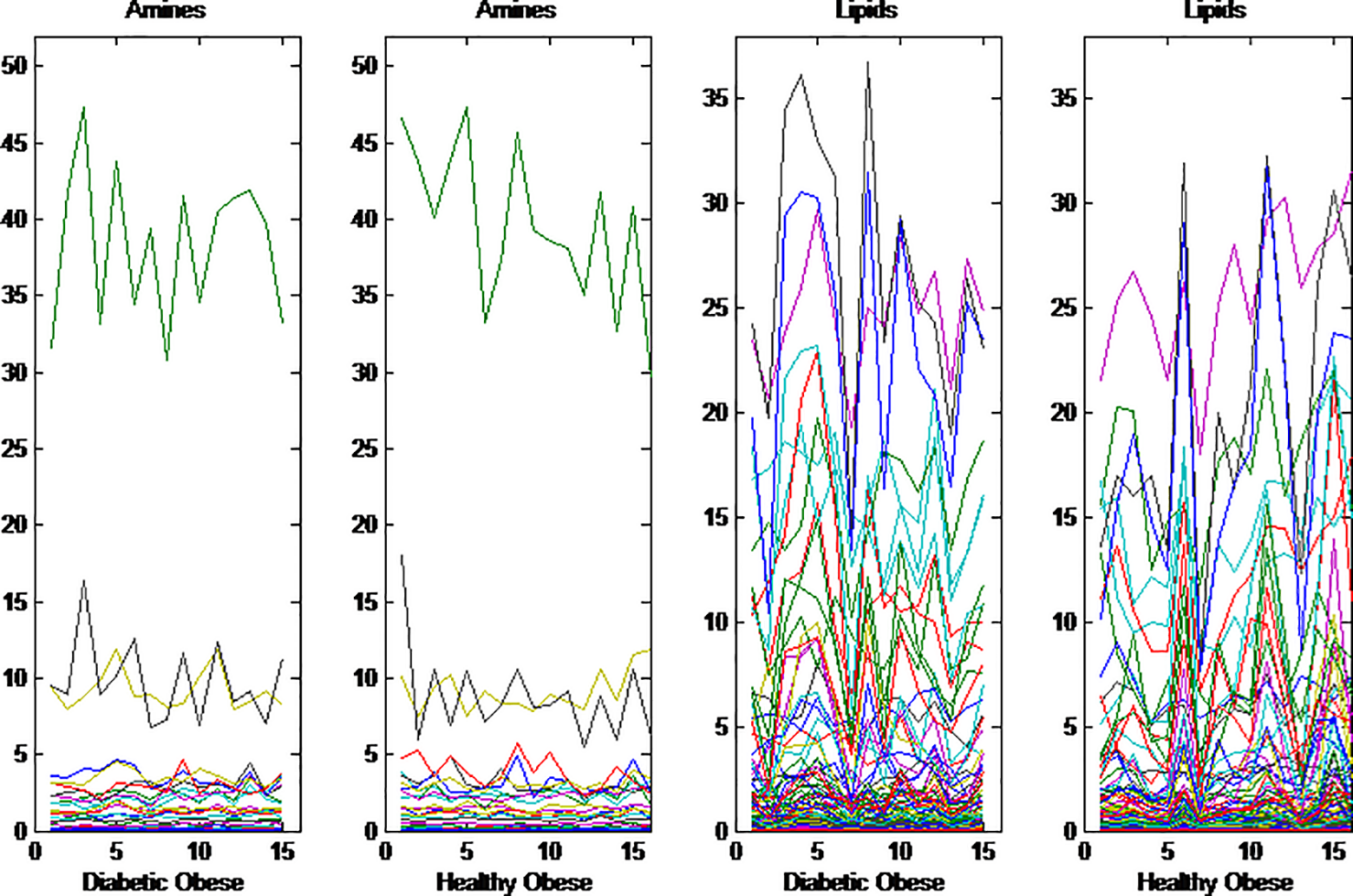
Amine and lipid levels. Amine levels and lipid levels for 15 Diabetic Obese individuals and 16 Healthy Obese individuals. Each color represents a different metabolite. The amine with large values for both groups is L-Glutamine.

Fig 2 shows the amino acids and lipid profiles of our selected samples; the 15 Diabetic Obese individuals and the 16 Healthy Obese individuals prior to a gastric bypass operation. In the amino acids data there are some metabolites with high levels; particularly L-glutamine has high levels compared to the other metabolites. A two-sided t-test per metabolite to see if there is a significant difference between the healthy obese and the obese with diabetes, shows that out of the 208 metabolites, there are 45 that have a p-value smaller than 0.05. Nine of them are amino acids and the other 36 are lipids. Only 2 metabolites are significant at a Bonferoni corrected limit for multiple testing, one amine and one lipid.

### 3.3. Effect of measurement error correction on simultaneous component analysis

#### 3.3.1. Transformation

**Fig 3:**
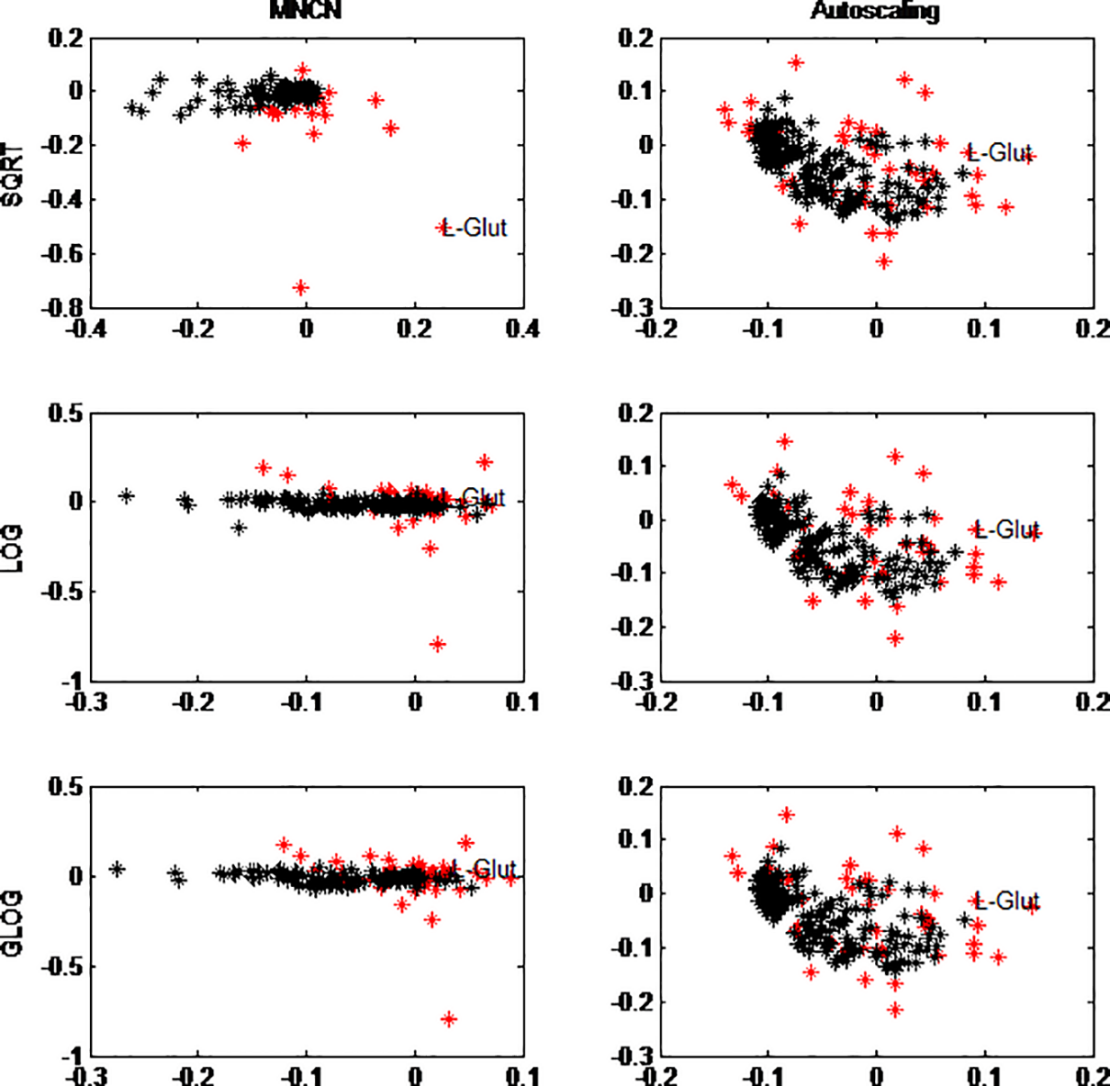
SCA loadings. SCA loading 1 (X-axis) and SCA loading 2 (Y-axis), for centered data (left column) or autoscaled data (right column) and additionally block-scaled after square root (SQRT) transformation (top row), LOG-transformation (middle row) and GLOG transformation (bottom row). The amino acids are indicated in red, black are the lipids. L-Glutamine (highly levelled amino acid) is indicated in all plots.

Fig 3 shows the first two loadings of the SCA after square root (SQRT), LOG and GLOG transformation. On the left the loadings of the mean-centered (after transformation) data are given and on the right the autoscaled (after transformation) data. Within the raw data, a scale effect of the variables could be seen; L-glutamine which had the highest values in the data also got the highest loadings (S1 Fig). This scale effect seems to be somewhat reduced by LOG- and GLOG-transformation of the data (Fig 3), i.e. L-glutamine which was an outlier in the raw data, is no longer an outlying metabolite. We would expect to see more metabolites with higher loadings, since 45 are significantly different using a two-sided t-test. After transformation this number even increases to 56 (12 amino acids), 59 (12 amino acids), and 60 (12 amino acids) for respectively LOG-, GLOG-, and SQRT transformation. Note that transformation does not change the order of the individuals for each metabolite, but it does change the within group variance. Since a normal t-test takes this into account, the results can change.

Autoscaling the data after transformation had a strong effect as this makes the SCA loadings almost independent of the transformation used. Comparing the autoscaled raw data loadings with the loadings of autoscaled data after the different transformation shows there is not much difference. Thus the effect of transformation on the loadings is small if autoscaling is used afterwards. This can be understood as the transformation does not change the order of the samples for a given metabolite, it only changes the distances between them, making those more equal. The autoscaling then centers the data and makes the variance of each metabolite equal. Only in cases of extreme heteroscedasticity, would one expect larger changes.

#### 3.3.2. Measurement error structures

**Fig 4:**
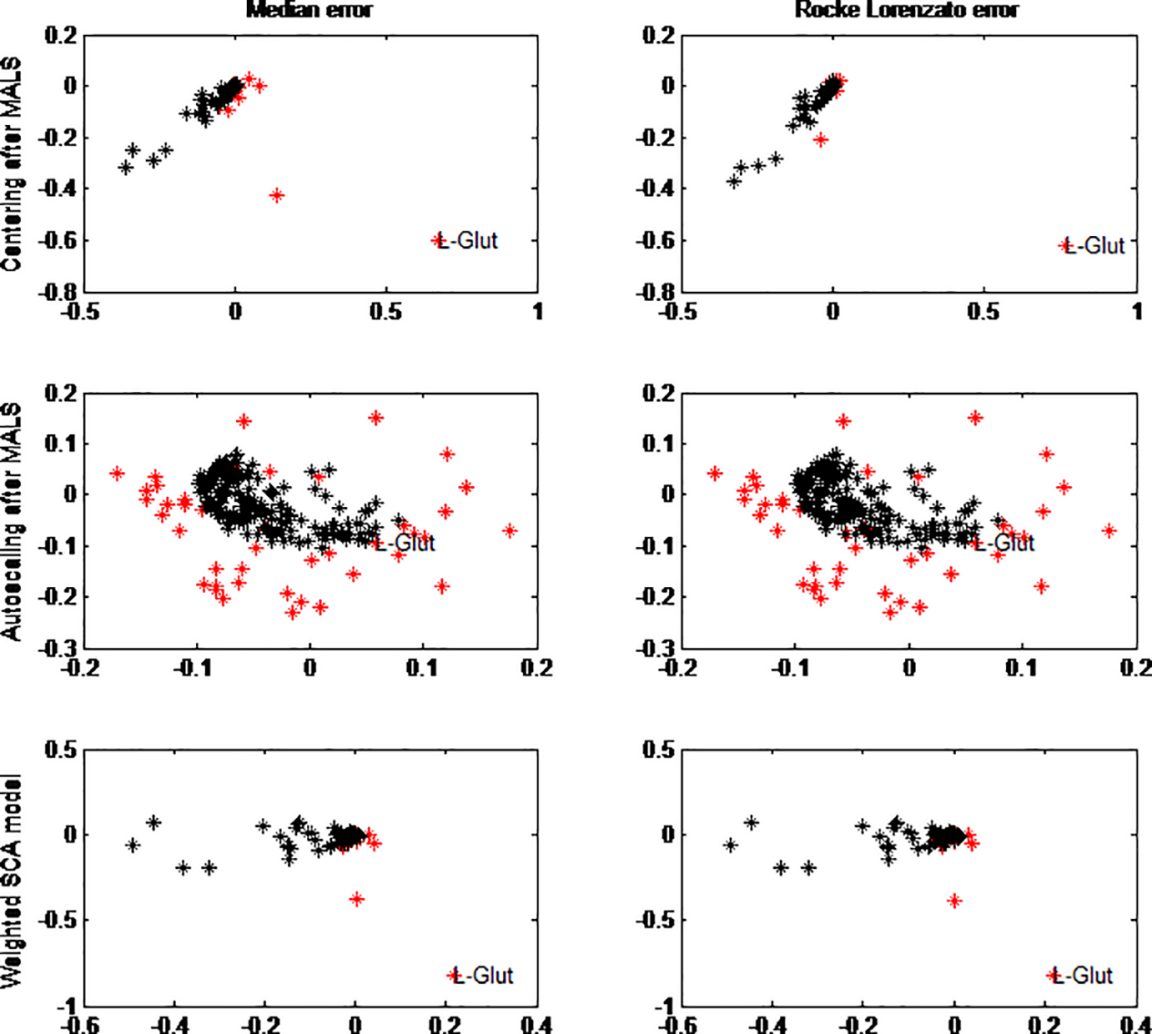
Effect of measurement error on SCA loading after MALS and weighted SCA model. On the X-axis is the loading of PC1 and on the Y-axis the loading of PC2. After MALS, centering (top row) and autoscaling (2^nd^ row) is applied. In the bottom row the loadings of the weighted SCA model are presented. In red are the amine loadings and in black the lipid loadings. Both the median error model (left column) and the Rocke Lorenzato error model (right column) are explored. L-Glutamine, the amine with large values is indicated in all plots.

Correction of unequal measurement error variance using filtering and modelling before and within the SCA model, respectively, with a maximum of 7 components was performed. The first two loadings are given in Fig 4. On the top row we see the loadings of the SCA of block scaled data after MALS filtering and centering for Median error structure (left) and Rocke-Lorenzato error structure (right). The difference in the two loading plots is very small, and the difference with the mean centered data without MALS (S1 Fig) is also small meaning that the MALS filtering does not lead to large differences in the cleaned data. Autoscaling after MALS (second row) has a large effect on the SCA loadings, but again the type of measurement error model (RL or Median) almost has no effect.

For the modelling method (maximum likelihood fusion), the loadings are similar to the loadings of the original SCA. This means that the effect of weighing the residuals does not have a large effect on the estimation of the scores and loadings. In these loading plots L-Glutamine is again the outlying metabolite. The weighted PCA cannot undo the large intensity differences in the data. Neither correction with median error structure nor correction with a Rocke-Lorenzato error structure corrects for the scale differences of the variable.

### 3.4. Effect of measurement error correction on simultaneous component discriminant analysis

The predictive performance of discrimination was studied, using 25 repeats of the cross validation with each time a different combination of test set samples. The number of components calculated for the training set was set to 3, 5, or 7 (W)PCDA components to study whether these models suffer from overfit. The number of components used in the MALS filter was set to 7. The repeats from which the weights were calculated are different work-ups of the same sample. The average number of misclassifications of the 25 cross-validations is given in Table 1. As expected, autoscaling is an important factor in almost all models; i.e. it decreases the number of misclassifications. When autoscaling is used, most approaches are already optimal with 5 components. When no autoscaling is used, in most cases more components are needed for lower classification error.

For the MALS filtering and the WSCDA modelling approach the number of misclassifications is generally higher than for the transformation methods. For the MALS and modelling approaches, no clear difference between the median error structure and the Rocke-Lorenzato error structure can be observed while a median error was expected to be a better model as it is calculated per metabolite.

Tables 2 and 3 give the top 10 of most influential metabolites on the discriminant performance of the different models, for 5 components. When no transformation has been used and no centering is applied then many of the glyceroPhosphoCholines and triglycerides are selected. After transformation and centering, the selected metabolites are different. Thus transformation has a clear effect on the selection of important variables. When autoscaling is used then L-Leucine, Glycine, N6N6N6trimethyllysine, D-L3aminoisobutyric acid, glutamic acid and others are selected. It can be seen that the selected variables are rather consistent when autoscaling is used after the transformation. Autoscaling is removing the difference that is applied by the different error variance stabilization approaches. Many of the same features are selected after autoscaling has been applied, independent of the transformation applied.

**Table 2:** Selected variables.

	Raw data		Transformation					
	Center	autoscaled	Sqrt + center	sqrt autoscaled	Log + Center	log + autoscaled	Glog + Center	Glog + Autoscaling
**1**	PC.36.2	L-leucine	Glycine	L-Leucine	3-Methyl histidine	L-Leucine	L-glutamic acid	L-Leucine
**2**	PC.34.2	Glycine	L-proline	Glycine	L-Glutamic acid	Glycine	L-alpha-aminobutyric acid	Glycine
**3**	PC.36.3	DL3aminoisobutyric acid	L-arginine	N6N6N6trimethyllysine	Citruline	N6N6N6trimethyllysine	3-Methyl histidine	N6N6N6trimethyllysine
**4**	TG.52.2.	L-arginine	L.glutamicacid	DL3aminoisobutyric acid	L-pipecolic acid	L-argenine	Glycine	L-argenine
**5**	L-lysine	L2-aminoadipicacid	L-Threonine	L-Arginine	Beta alanine	L2-aminoadipicacid	Citruline	L2-aminoadipicacid
**6**	TG.50.1	Epinephrine	PC.36.2	L2-aminoadipicacid	Glycine	3-Methyl histidine	L-argenine	3-Methyl histidine
**7**	L-Arginine	N6N6N6trimethyllysine	3-Methylhistidine	3-methylhistidine	L-methionine sulfoxide	DL3aminoisobutyric acid	TG.58.1	DL3aminoisobutyric
**8**	L-Proline	L-glutamic acid	PC.36.3	epinephrine	L-alpha-aminobutyric acid	epinephrine	DL3aminoisobutyric acid	L.glutamicacid
**9**	SM.d18.1.16.0.	3-Methylhistidine	Citruline	L-glutamic acid	L-Arginine	L.glutamicacid	L2-aminoadipicacid	epinephrine
**10**	TG.52.1	Beta-alanine	L-glutamine	Beta-alanine	LPE2.26	L-valine	TG.48.4	L-valine

Top 10 of selected variables in the 25 cross validation models, with 5 principal components for the transformation methods

**Table 3:** Selected variables for MALS methods.

	MALS RL		MALS MEdian		Modeling	
	Center	Autoscaled	Center	Autoscaled	Median	RL
**1**	TG5.22	Taurine	L-proline	Taurine	PC.36.2	PC.36.2
**2**	PC.34.2	DL3aminoisobutyric acid	TG.52.2	3-methylhistidine	PC.34.2	PC.34.2
**3**	PC.36.2	L-leucine	TG.52.3	N6N6N6trimethyllysine	L-proline	L-proline
**4**	PC.36.3	N6N6N6trimethyllysine	PC.36.4	PCO.36.2	PC.36.3	PC.36.3
**5**	TG.52.3.	L-argenine	TG.50.1	TG.55.1	TG.52.2	TG.52.2
**6**	L-proline	Glycine	Glycine	TG.52.1	PC.36.4	PC.36.4
**7**	PC.34.1	L-Valine	PC.50.2	TG.57.1	SM.d18.12.31	SM.d18.12.31
**8**	TG.50.1	L-alpha-aminobutyric acid	L-argenine	SM.d18.12.31	TG.50.2	TG.50.2
**9**	TG.50.2	L-Threonine	L-serine	CE.18.3	TG.52.3	PC.38.4
**10**	SM.d18.1.16.0.	L-kynurenine	L-threonine	L-Isoleucine	PC.38.4	TG.52.3

Table 3: The top 10 number of selected variables in the 25 cross-validation models, with 5 principal components for the filtering (MALS) and modeling methods.

### 3.5. Selected metabolites

When autoscaling is used then glycine and the branched chain amino acids (BCAA) L-leucine and L-valine are often selected. Glycine has recently been found to have the strongest (positive) correlation with insulin sensitivity of all amino acids, even stronger than the (negative) correlation with branched chain amino acids, leucine, isoleucine and valine [26]. This study was performed in subjects with BMI averaging 30–33, which is well below the average BMI in our subjects of >40 [27]. Since the inclusion criterion for our T2DM subjects was an increased fasting plasma glucose level (>7 mmol/l), which is a surrogate measure for insulin sensitivity, this indicates that the correlation of insulin sensitivity with glycine may extend to subjects with extreme overweight and T2DM. At present, it is unknown whether glycine is causally related to insulin action. Glutamic acid is both selected in the model with and without autoscaling. Glutamic acid and glycine are both neurotransmitters. Whether increased plasma levels reflect changes in, or affect central and peripheral signaling is not known. Phosphatidylcholine (PC) 36:2 is one of the species of PCs that are structural components of plasma membranes and the surface of lipid droplets. In plasma, PCs are present on circulating membrane fragments (micro-particles) and lipoproteins. To what extent the observed changes in levels of PC36:2 affect biological functions of membrane fragments and lipoproteins is not known.

## Discussion

We have shown that simultaneous component analysis can successfully be incorporated into principal component discriminant analysis to provide a good tool to investigate underlying relationships in metabolomics data obtained through multiple platforms. To better understand the bottlenecks involved in integrating multiple platforms of metabolomics data, we have studied how relevant error structures can be determined, and what the effect is of different error models, via different ways.

Autoscaling of raw and transformed data improves the predictive performance of the SCDA models. Furthermore, fewer components are necessary for models on autoscaled data, and the models are more stable. It seems that biological variation within the samples, i.e. scale-size differences, have a large influence on the predictive performance. When this is neglected, the loadings are proportional to the variance, and can possible influence the classification performance. This scale-size effect is not solved by correcting for the measurement errors. Although for models with large numbers of components, weighted SCA performs equally well as the autoscaled raw/transformed data.

Data transformations are easily adapted methods to deal with increasing measurement error. LOG-transformation usually overcorrects the errors for small ratios, while GLOG assumes a constant error variance for smaller ratios and an increasing error variance for bigger ratios [13]. In our data, it seems that GLOG-transformation also increases the error for small intensities. Moreover, all three transformation methods seem to perform quite similar in the predictive performance and the number of selected components. There seems to be a small decrease in the number of misclassifications after transformation of the data, especially the mean-centered square root transformed data has a lower number of misclassifications compared to the raw mean-centered data, although it tends to select a higher number of components.

The error structures are obtained from the sample replicates, both the sample replicates from paired data (7 sample replicates) as well as from the other individuals with replicated samples (8 sample replicates).

The maximum likelihood fusion is incorporated in the SCDA, in the sense that contrary to MALS it does not stabilize the measurement error variance of the data beforehand. In MALS, the first step is to filter out noise by using weighted PCA, where each data block is optimized separately, after that the data is fused and the SCDA is performed on this noise-free fused data. In maximum likelihood fusion the data is fused and afterwards optimized on the number of components that give the best predictive performance, and not as in MALS to get the best error-free data. Maximum likelihood fusion might therefore be suboptimal, especially in models where the maximum number of principal components is small.

Correction using the Rocke-Lorenzato error structure per lipids class and all amino acids seems to be less effective compared to the median error structure per metabolite, especially for models with fewer components and within the MALS procedure. It seems that an error structure per metabolite gives a more precise prediction of the underlying error structure than an error structure per groups of metabolites. Van Batenburg et al.[11] argued that due to different error sources, each metabolite can have a different error structure, therefore grouping metabolites to determine error structures might underestimate the real complexity of the data.

For an easier understanding of the model, an SCDA model with fewer components is preferred over a model with more components. Moreover, to reduce computation time, the maximum number of principal components should be kept low, nevertheless, one should be careful not to select too few components, as for non-autoscaled methods, the filtering method, and the modelling method, a smaller number of principal components means a less exact class prediction.

Weighted PCA, either in the MALS or in the maximum likelihood fusion, does not seem to contribute a lot to the predictive performance of the model obtained via simultaneous component discriminant analysis (SCDA). Faster methods, like square root-, LOG-, and GLOG-transformation perform equally well. Moreover, square root- and (G)LOG -transformations do not even require sample replicates. However, an important step in class prediction via SCDA, additional to potential transformation, is to remove any scale-size effects between variables via autoscaling.

## Supporting information

S1 Fig SCA loadings of RAW data after mean centering or autoscaling and block scaling to equal Frobenius norm. The red markers indicate loadings of amines while the black markers are of lipid metabolites. L-Glutamine is indicated because of its high values in the raw data. To ensure that the analysis is not completely driven by one metabolite with large values, we examined the effect of autoscaling on the loadings of the SCA. For this, SCA was performed on mean centered block scaled raw data and autoscaled block scaled raw data. The first two principal loadings are plotted in S1 Fig. In the mean centered analysis, L-glutamine gets high loadings, and most likely will drive the analysis. Though, L-glutamine is not-significantly different between the two classes (double sided t-test p-value = 0.4958). Autoscaling the variables seems to be a necessary step to make sure that extreme metabolites do not drive the analysis; autoscaling makes the loadings more comparable with each other, such that the scale of the variables no longer has an influence on the analysis (see S1 Fig right).
(TIF)

S1 File Amines. The file amines.csv contains the Amine metabolite levels of the patient samples.
(CSV)

S2 File QC corrected Amine levels. The file amine_qc.csv contains the amine metabolite levels of the patient samples after QC correction.
(CSV)

S3 File Lipids. The file Lipids.csv contains the lipid levels of the patient samples.
(CSV)

S4 File QC corrected Lipids. The file lipids_qc.csv contains the lipid levels of the patient samples after QC correction.
(CSV)

S5 File Patient sample information. The file NMC0913groepen.csv contains information on the patient samples.
(CSV)

## References

[1] Christin C, Hoefsloot HCJ, Smilde AK, Hoekman B, Suits F, Bischoff R, et al A critical assessment of feature selection methods for biomarker discovery in clinical proteomics. *Molecular & Cellular Proteomics*. 2013;12(1):263–276. 23115301 10.1074/mcp.M112.022566 PMC3536906

[2] Hoogerbrugge R, Willig SJ, Kistemaker PG. Discriminant-analysis by double stage principal component analysis. *Anal Chem*. 1983;55(11):1710–1712.

[3] Barker M, Rayens W. Partial least squares for discrimination. *Journal of Chemometrics*. 2003;17(3):166–173.

[4] Lutz U, Lutz RW, Lutz WK. Metabolic profiling of glucuronides in human urine by LC-MS/MS and partial least-squares discriminant analysis for classification and prediction of gender. *Analytical Chemistry*. 2006;78(13):4564–4571. doi: 10.1021/ac0522299 16808466 10.1021/ac0522299

[5] Szymanska E, Saccenti E, Smilde AK, Westerhuis JA. Double-check: Validation of diagnostic statistics for PLS-DA models in metabolomics studies. *Metabolomics*. 2012;8(1):S3–S16. 10.1007/s11306-011-0330-3 PMC3337399 22593721

[6] Van Deun K, Wilderjans TF, van den Berg RA, Antoniadis A, Van Mechelen I. A flexible framework for sparse simultaneous component based data integration. *BMC Bioinformatics*. 2011;12:448 doi: 10.1186/1471-2105-12-448 22085701 10.1186/1471-2105-12-448 PMC3283562

[7] Wilderjans TF, Ceulemans E, Van Mechelen I, van den Berg RA. Simultaneous analysis of coupled data matrices subject to different amounts of noise. *British Journal of Mathematical & Statistical Psychology*. 2011;64(2):277–290. 21492133 10.1348/000711010X513263

[8] van den Berg RA, Van Mechelen I, Wilderjans TF, Van Deun K, Kiers HAL, Smilde AK. Integrating functional genomics data using maximum likelihood based simultaneous component analysis. *BMC Bioinformatics*. 2009;10:340 doi: 10.1186/1471-2105-10-340 19835617 10.1186/1471-2105-10-340 PMC2771021

[9] Parsons HM, Ekman DR, Collette TW, Viant MR. Spectral relative standard deviation: A practical benchmark in metabolomics. *Analyst*. 2009;134(3):478–485. doi: 10.1039/b808986h 19238283 10.1039/b808986h

[10] Rocke D, Lorenzato S. A 2-component model for measurement error in analytical-chemistry. *Technometrics*. 1995;37(2):176–184.

[11] Van Batenburg MF, Coulier L, van Eeuwijk F, Smilde AK, Westerhuis JA. New figures of merit for comprehensive functional genomics data: The metabolomics case. *Anal Chem*. 2011;83(9):3267–3274. doi: 10.1021/ac102374c 21391558 10.1021/ac102374c

[12] van den Berg RA, Hoefsloot HCJ, Westerhuis JA, Smilde AK, van der Werf MJ. Centering, scaling, and transformations: Improving the biological information content of metabolomics data. *BMC Genomics*. 2006;7:142 doi: 10.1186/1471-2164-7-142 16762068 10.1186/1471-2164-7-142 PMC1534033

[13] Purohit PV, Rocke DM, Viant MR, Woodruff DL. Discrimination models using variance-stabilizing transformation of metabolomic NMR data. *Omics-a Journal of Integrative Biology*. 2004;8(2):118–130. doi: 10.1089/1536231041388348 15268771 10.1089/1536231041388348

[14] Hoefsloot HCJ, Verouden MPH, Westerhuis JA, Smilde AK. Maximum likelihood scaling (MALS). *J Chemometrics*. 2006;20(3–4):120–127.

[15] Hoefsloot HCJ, Smit S, Smilde AK. A classification model for the leiden proteomics competition. *Statistical Applications in Genetics and Molecular Biology*. 2008;7(2):8. 10.2202/1544-6115.1351 18312222

[16] Timmerman ME, Kiers HAL. Four simultaneous component models for the analysis of multivariate time series from more than one subject to model intraindividual and interindividual differences. *Psychometrika*. 2003;68(1):105–121.

[17] Ten Berge J, Kiers HAL, Stel V. Simultaneous component analysis. *Statistica Applicata*. 1992;4:377.

[18] Timmerman ME. Multilevel component analysis. *British Journal of Mathematical & Statistical Psychology*. 2006;59:301–320. 17067414 10.1348/000711005X67599

[19] Westerhuis JA, Kourti T, MacGregor JF. Analysis of multiblock and hierarchical PCA and PLS models. *J Chemometrics*. 1998;12(5):301–321.

[20] Durbin BP, Hardin JS, Hawkins DM, Rocke DM. A variance-stabilizing transformation for gene-expression microarray data. *Bioinformatics*. 2002;18 Suppl 1:S105–10. 12169537 10.1093/bioinformatics/18.suppl_1.s105

[21] Parsons HM, Ludwig C, Guenther UL, Viant MR. Improved classification accuracy in 1-and 2-dimensional NMR metabolomics data using the variance stabilising generalised logarithm transformation. *BMC Bioinformatics*. 2007;8:234 doi: 10.1186/1471-2105-8-234 17605789 10.1186/1471-2105-8-234 PMC1965488

[22] Durbin B, Rocke DM. Estimation of transformation parameters for microarray data. *Bioinformatics*. 2003;19(11):1360–1367. 12874047 10.1093/bioinformatics/btg178

[23] Bro R, Sidiropoulos N, Smilde A. Maximum likelihood fitting using ordinary least squares algorithms. *J Chemometrics*. 2002;16(8–10):387–400.

[24] van der Kloet FM, Bobeldijk I, Verheij ER, Jellema RH. Analytical error reduction using single point calibration for accurate and precise metabolomic phenotyping. *Journal of Proteome Research*. 2009;8(11):5132–5141. doi: 10.1021/pr900499r 19754161 10.1021/pr900499r

[25] The Mathworks Inc. MATLAB version 7.10.0., 2010.

[26] Thalacker-Mercer AE, Ingram KH, Guo F, Ilkayeva O, Newgard CB, Garvey WT. BMI, RQ, diabetes, and sex affect the relationships between amino acids and clamp measures of insulin action in humans. *Diabetes*. 2014;63(2):791–800 doi: 10.2337/db13-0396 24130332 10.2337/db13-0396 PMC3900549

[27] Lips M.A., van Klinken J.B., van Harmelen V., Dharuri H.K., ‘t Hoen P.A, Laros J.P., et al Roux and Y gastric bypass surgery, but not calorie restriction, reduces plasma branched chain amino acids in obese subjects independent of weight loss or the presence of type 2 diabetes mellitus. Diabetes Care. 2014 37:3150–6. doi: 10.2337/dc14-0195 25315204 10.2337/dc14-0195

