# Supplementary figures and images for: Fusing metabolomics data sets with heterogeneous measurement errors
Source: PLoS One. 2018 Apr 26;13(4):e0195939. doi: 10.1371/journal.pone.0195939 (PMC5919515; doi:10.1371/journal.pone.0195939)

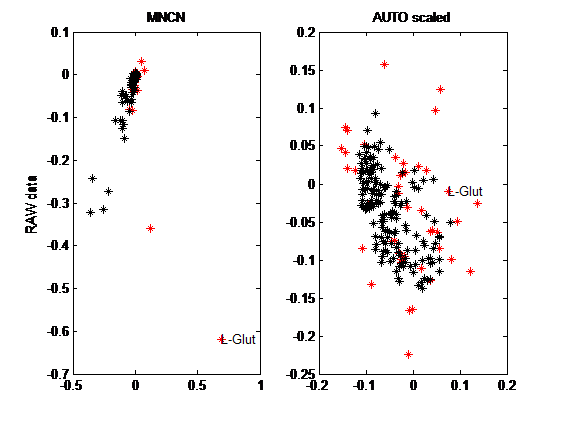

Supplement: S1 Fig — The red markers indicate loadings of amines while the black markers are of lipid metabolites. L-Glutamine is indicated because of its high values in the raw data. To ensure that the analysis is not completely driven by one metabolite with large values, we examined the effect of autoscaling on the loadings of the SCA. For this, SCA was performed on mean centered block scaled raw data and autoscaled block scaled raw data. The first two principal loadings are plotted in S1 Fig. In the mean centered analysis, L-glutamine gets high loadings, and most likely will drive the analysis. Though, L-glutamine is not-significantly different between the two classes (double sided t-test p-value = 0.4958). Autoscaling the variables seems to be a necessary step to make sure that extreme metabolites do not drive the analysis; autoscaling makes the loadings more comparable with each other, such that the scale of the variables no longer has an influence on the analysis (see S1 Fig right). (TIF) [file pone.0195939.s001.tif]
